# Swirl sign in intracerebral haemorrhage: definition, prevalence, reliability and prognostic value

**DOI:** 10.1186/1471-2377-12-109

**Published:** 2012-09-26

**Authors:** Eufrozina Selariu, Elisabet Zia, Marco Brizzi, Kasim Abul-Kasim

**Affiliations:** 1 Neuroradiology Division, Diagnostic Centre for Imaging and Functional Medicine, Lund University, Skåne University Hospital, Malmö, 205 02, Sweden; 2 Department of Neurology, Lund University, Skåne University Hospital, Malmö, 205 02, Sweden

**Keywords:** Swirl sign, Computed tomography, Reliability, Functional outcome, Haemorrhage volume

## Abstract

**Background:**

Swirl sign has previously been described in epidural hematomas as areas of low attenuation, radiolucency or irregular density. The aims of this study were to describe swirl sign in ICH, study its prevalence, study the reliability of the subjective evaluation on computed tomography (CT), and to explore its prognostic value.

**Methods:**

CTs of 203 patients with ICH were retrospectively evaluated for the presence of swirl sign. Association between swirl sign and different clinical and radiological variables was studied.

**Results:**

Inter- and intraobserver agreement with regard to the occurrence of swirl sign was substantial (К 0.80) and almost perfect (К 0.87), respectively. Swirl sign was found in 30% of the study population. 61% of patients with swirl sign were dead at one month compared with 21% of those with no swirl sign (p < 0.001). Only 19% of patients with swirl sign exhibited favorable outcome at three months compared with 53% of those with no swirl sign (p < 0.001). Patients with swirl sign exhibited larger ICHs with average ICH-volume 52 ± 50 ml (median 42 ml) compared with 15 ± 25 ml (median 6) in patients whose CT did not show swirl sign (p < 0.001). Swirl sign was independent predictor of death at one month (p = 0.03; adjusted odds ratio 2.6, 95% CI 1.1 – 6), and functional outcome at three months (p = 0.045; adjusted odds ratio 2.6, 95% CI 1.02 – 6.5).

**Conclusions:**

As swirl sign showed to be an ominous sign, we recommend identification of this sign in cases of ICHs.

## Background

One-month case fatality rate among patients with intracerebral haemorrhage (ICH) is high, 25–50% [1,2]. Haemorrhage volume is an important prognostic factor [3] and in one third of patients with ICH, there is an expansion of the hematoma volume during the first hours after ictus [4]. Identification of patients with ICH with a potential risk for hematoma expansion is crucial for planning of the treatment and prediction of the functional outcome. With the advent of computed tomography angiography (CTA) in the last decade, spot sign or contrast extravasation has been shown to be an independent predictor of hematoma expansion [5]. However, CTA requires iodine contrast administration that might be harmful in older patients especially those with impairment of renal function. Therefore, scrutinizing plain CT for other possible signs that might have prognostic value is worthwhile for patients with potential contraindications to contrast administration.

On non-enhanced computed tomography (CT), swirl sign was originally described as areas of low attenuation or radiolucency inside intracranial hyperattenuated hematomas [6,7]. Zimmerman et al. have studied swirl sign in 45 epidural hematomas and concluded that this sign indicates active bleeding that warrant immediate surgical treatment [8]. In another report, 13 patients with acute subdural and epidural hematoma found to have fresh non-clotted blood upon surgical exploration and in 11 of them active bleeding foci were identified [9].

The utility of swirl sign in ICH has not been studied in depth. The aims of this study were to (a) describe swirl sign and its different imaging varieties in ICH, (b) study its prevalence among patients with ICH, (c) explore the subjective inter- and intraobserver reliability in evaluating this sign on non-enhanced CT, and finally (d) find out if the occurrence of this sign has any prognostic value.

## Methods

This study included patients with spontaneous ICH admitted to our university hospital, between January 2007 and June 2009. Our hospital is the only one serving the population of about 300 000. Patients with ICD code I10.1-9 were identified from the National Hospital Discharge Register [10]. A research nurse, neurologists and a neuroradiologist validated the diagnosis by reviewing the medical records and CTs of these patients, respectively.

Information about diabetes (diet or pharmacological treatment), treatment with antithrombotics (Aspirin, Clopidogrel, Dipyridamole), warfarin treatment, hypertension medication, smoking habit (current smoker/non smoker) on admission, and the time between onset of stroke and arrival to hospital, was retrieved from patient medical journal. Level of consciousness on admission was assessed according to the Reaction Level Scale (RLS 85 scale) [11,12]. The RLS scores were categorized into three groups: RLS score = 1 (alert), score 2–3 (drowsy) and score ≥ 4 (unconscious).

All patients included in this study were subjected to non-enhanced head CT on admission. All examinations were performed at 16 slice multidetector CT-scanner (SOMATOM 16 Sensation, Siemens AG, Forchheim, Germany). Axial 4.5 mm thick slices and reconstructed 3 mm coronal images were used for the evaluation in the Picture Archiving and Communication System (PACS, SECTRA). 0.75 mm thick slices were also available in PACS enabling further multiplanar reconstruction (MPR) when needed. The images were evaluated with regard to: (a) Swirl sign, (b) side and site of the ICH, (c) presence of midline shift, (d) intraventricular haemorrhage, (e) presence of white matter changes according to Wahlund score [13], and (f) haemorrhage volume. The maximum haemorrhage width (W, transverse diameter), length (L, anteroposterior diameter) and height (H, craniocaudal diameter) were measured and the haemorrhage volume was estimated using the following formula (W x L x H x 0.5). Swirl sign was evaluated independently by 2 neuroradiologists (KAK, ES) experienced in stroke imaging. At the time of evaluation of swirl sign, the readers were blinded to the clinical data. One reader (KAK) has performed the same evaluation at two different occasions with 8- weeks interval.

**Figure 1:**
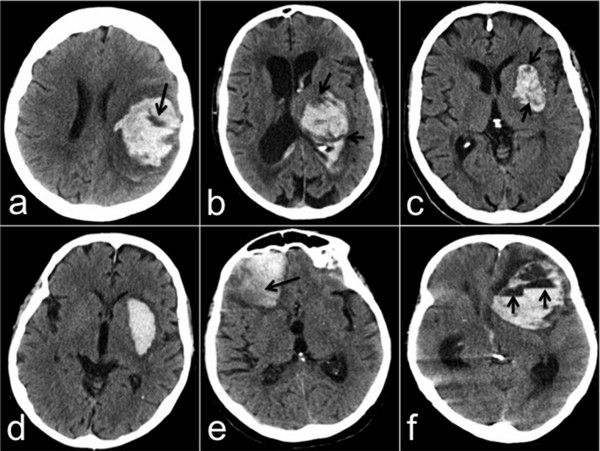
**Different examples of swirl sign and swirl sign mimics.** Axial images of 6 different patients with ICH. (**a**–**c**) Show different forms of swirl sign; well defined in a, irregular in b, and streak-like in **c**. (**d**) Shows homogeneously hyperattenuated left-sided putaminal haemorrhage with no swirl sign. (**e**) Shows right-sided subacute frontal haemorrhage with lower attenuation (black arrow) at the outer posterior portion indicating beginning resorption (clot retraction) that should not be addressed as swirl sign. (**f**) Shows left-sided subacute frontal haemorrhage with multiple blood-fluid levels (short arrows) that should not be addressed as swirl sign.

Swirl sign was defined as region(s) of hypoattenuation or isoattenuation (compared to the attenuation of brain parenchyma) within the hyperattenuated ICH. The areas of hypoattenuation or isoattenuation may vary in shape and can be rounded, streak-like or irregular, Figure 1. The sign should be observed on both axial and coronal plane. In cases where readers disagreed about the presence of swirl sign, performing joint panel evaluation by the two readers made the final decision about the occurrence of swirl sign.

Information about the date of death was assessed from the National Cause of Death Register [14]. All patients with validated stroke diagnosis at the hospital are routinely registered in a National Stroke Register [15]. Modified Rankin Scale (mRS) [16] at three months as a measure of functional outcome was achieved from the three months follow-up questionnaire in this register. Eriksson et al. created a key to convert those self-reported answers into mRS [17]. Upon analysis of the functional outcome, patients with mRS of ≤ 3 were considered to have favorable outcome whereas patients with mRS ≥ 4 were considered to have unfavorable outcome.

This project was approved by the Regional Ethical Review Board- Lund, Sweden (LU 238–02).

### Statistical analysis

All statistical analyses were performed by means of Statistical Package for the Social Sciences (SPSS) version 18. Data is presented as proportions (%) or as mean ± standard deviations (SD). The degree of interobserver and intraobserver agreement with regard to the occurrence of swirl sign was estimated by cross tabulation and calculation of kappa (К-value). The interpretation of kappa values was done according to the one proposed by Landis [18]. A kappa of 1 indicates total agreement whereas a kappa of zero means poor agreement and indicates that any observed agreement is attributed to chance. Mann Whitney-U test has been used to test the association between categorical and continuous variables, and chi-square test for the association between categorical variables. Logistic regression analysis was performed to find out if swirl sign was an independent predictor of death at one month and functional outcome at three months. Variables with p < 0.05 in univariate analysis were considered as potential confounders and included in the final model.

## Results

### Characteristics of study cohort

This study included 203 patients with spontaneous ICH with mean age of 73 ± 14 years (range 29–97); 112 (55%) were females. The mean age of patients whose initial CT showed swirl sign was the same as that of patients with no swirl sign (73 years).

### Inter- and intraobserver reliability

The interobserver agreement (i.e. the agreement between the two readers that performed the evaluation of CTs with regard to the occurrence of swirl sign) was substantial (К- value of 0.80; 95% CI 0.71 – 0.89) whereas the intraobserver agreement (i.e. the agreement between the assessment done by the reader who performed the same evaluation at two different occasions) was almost perfect (К- value of 0.87; 95% CI 0.80 – 0.94).

In 14 out of 203 patients, the two readers were not agreed about the status of swirl sign at any of the three occasions of evaluation. Four out of these 14 patients had small bleedings with a volume ≤ 5 ml. The final decision about the occurrence of swirl sign was made by joint evaluation of the two readers to reach a consensus.

### Prevalence of swirl sign in study population

Swirl sign was found on the non-enhanced CT of 61 (30%) out of 203 patients with ICH. In ICHs with haemorrhage volume 5–30 ml, the incidence of swirl sign was 41% (57 out of 138 patients) compared with 62% in patients with haemorrhage volume ≥ 30 ml (33 out of 53 patients), p = 0.010. Of 65 with haemorrhage volume 1–4 ml, only 4 (6%) showed swirl sign.

### Association of swirl sign with different clinical variables

There was no significant association between the occurrence of swirl sign and the gender, diabetes mellitus, history of former stroke, smoking or treatment with platelet activator inhibitor or Warfarin. Of 128 patients with antihypertensive treatment at admission, CTs of only 30 patients (23%) showed swirl sign compared to 31 out of 74 (42%) patients with no antihypertensive treatment (p = 0.005), Table 1. Regarding the level of consciousness upon arrival to hospital, 19 out of 35 patients (54%) with RLS ≥ 4 showed swirl sign on the initial CT compared with 19 out of 99 patients (19%) with RLS 1 (p < 0.001), Table 1. Of 88 patients who arrived to the emergency department within 2 h from symptom onset, 32 (36%) showed swirl sign compared with 4 out of 31 (13%) patients who arrived after 24 h (p = 0.051), Table 1.

**Table 1:** The association between the occurrence of swirl sign and different variables included in the analysis of this study

		**Swirl sign**			**OR (95% CI)**	**p-value**
		**Negative**	**Positive**	**Total**		
Gender	Male	64 (70)	27 (30)	91		
	Female	78 (70)	34 (30)	112	1 (0.5-2)	0.520
Diabetes mellitus	Yes	17 (74)	6 (26)	23		
	No	125 (69)	55 (31)	180	1.3 (0.4-3.8)	0.431
Former stroke (1)	Yes	30 (77)	9 (23)	39		
	No	111 (68)	52 (32)	163	1.6 (0.7-3.8)	0.189
Smoking (5)	Yes	16 (70)	7 (30)	23		
	No	123 (70)	52 (30)	175	1 (0.4-2.8)	0.558
Hypertension treatment on admission (1)	Yes	98 (77)	30 (23)	128		
	No	43 (58)	31 (42)	74	2.4 (1.2-4.6)	**0.005**
Acetyl salicylic acid treatment (11)	Yes	22 (59)	15 (41)	37		
	No	112 (72)	43 (28)	155	0.6 (0.3-1.3)	0.306
Warfarin treatment (9)	Yes	12 (67)	6 (33)	18		
	No	123 (70)	53 (30)	176	0.9 (0.3-2.7)	0.483
Platelet activator inhibitor treatment (11)	Yes	25 (61)	16 (39)	41		
	No	109 (72)	42 (28)	151	0.6 (0.3-1.3)	0.117
Time to hospital (6)	< 2 h	56 (64)	32 (36)	88		
	2-24 h	54 (69)	24 (31)	78		
	> 24 h	27 (87)	4 (13)	31	0.3 (0.1-0.9)	0.051
RLS (5)	1	80 (81)	19 (19)	99		
	2 – 3	41 (64)	23 (36)	64		
	≥ 4	16 (46)	19 (54)	35	5 (2–12,6)	**<0.001**
Side	Left	68 (67)	34 (33)	102		
	Right	74 (73)	27 (27)	101	0.7 (0.4-1.4)	0.192
Location	Lobar	55 (67)	27 (33)	82		
	Deep	66 (73)	25 (27)	91		
	Brain stem	9 (82)	2 /18)	11		
	Cerebellum	11 (65)	6 (35)	17		
	Multiple	1(50)	1 (50)	2	0.8 (0.4-1.6)	0.745
Midline shift	Yes	23 (43)	30 (57)	53		
	No	119 (79)	31 (21)	150	0.2 (0.1-0.4)	**<0.001**
Intraventricular hemorrhage	Yes	35 (51)	33 (49)	68		
	No	107 (79)	28 (21)	135	0.3 (0.1-0.6)	**<0.001**
White matter lesions, Wahlund score	0	34 (65)	18 (35)	52		
	1	44 (75)	19 (25)	63		
	2	25 (71)	10 (29)	35		
	3	39 (74)	14 (26)	53	0.7 (0.3-1.7)	0.118

Figures between parentheses in the first column show the number of the missing data in each of the studied variables.

Figures between parentheses under swirl sign (column 3 and 4) show percentage.

p-values written in bold are statistically significant.

*RLS* reaction level scale, *OR* Odds ratio. 95%, *CI* 95% confidence interval.

### Association of swirl sign with different radiological variables

Out of 203 patients, 102 had ICH on the left side. 91 patients (45%) had deep haemorrhage and 82 (40%) had lobar haemorrhage, Table 1. None of the above variables was significantly associated with the occurrence of swirl sign, Table 1. The average haemorrhage volume among patients whose initial CT showed swirl sign was 52 ± 50 ml (median 42 ml) compared with 15 ± 25 ml (median 6) in patients whose CT did not show swirl sign (p < 0.001). 30 out of 61 (49%) patients with swirl sign exhibited midline shift, whereas among 142 patients with no swirl sign, only 23 (16%) exhibited midline shift (p < 0.001). Intraventricular haemorrhage occurred among 33 out of 61 patients (54%) with swirl sign compared with 35 (25%) among 142 patients whose initial CT did not show swirl sign (p < 0.001) Table 1.

### Death and functional outcome

Out of 61 patients with swirl sign, 37 (61%) were dead at one month compared with 29 out of 141 (21%) whose CT did not show swirl sign (P < 0.001), Table 2. Data on mRS were available on 188 out of 203 patients, 59 (31%) showed swirl sign on initial CT. Only 11 of 59 (19%) patients with swirl sign exhibited favorable outcome at three months (mRS ≤ 3). Among patients whose initial CT did not show swirl sign, 53% (69 out of 129) showed favorable outcome at three months (P < 0.001), Table 2.

**Table 2:** The association between the occurrence of swirl sign and death at one month and the functional outcome according to mRS

		**Swirl sign**		**OR (95% CI)**	**p-value**
		**Negative**	**Positive**		
Death one month*	Yes	29 (44)	37 (56)		
	No	112 (82)	24 (18)	0.2 (0.1-0.3)	< 0.001
mRS**	Favorable	69 (86)	11 (14)		
	Unfavorable	60 (56)	48 (44)	5 (2.3-11.3)	< 0.001

Figures between parenthesis are percentage.

* The number of the missing data is 1 patient. ** The number of the missing data is 15 patients.

*mRS* Modified Rankin Scale, favorable outcome: mRS ≤ 3, unfavorable outcome: mRS ≥ 4.

*OR* Odds ratio. 95%, *CI* 95% confidence interval.

After adjustment for potential confounders, logistic regression analysis showed that swirl sign was an independent predictor of death at one month (p = 0.03; adjusted odds ratio 2.6, 95% CI 1.1 – 6), and functional outcome at three months (p = 0.045; adjusted odds ratio 2.6, 95% CI 1.02 – 6.5).

## Discussion

This study showed that the subjective evaluation of swirl sign on non-enhanced CT is a reliable method. Swirl sign was found in 30% of our study population of 203 patients with ICH. Already in 1976, New et al. studied the CT attenuation of different blood components and concluded that extravasated blood may have attenuation levels within the range of attenuation of normal brain and an acute intraaxial haemorrhage might appear less dense than the brain [19]. Swirl sign was reported among 58% (26 out of 45) patients with of epidural hematomas and upon surgical exploration of 25 patients, 23 patients (92%) showed evidence of active bleeding [8].

Barras et al. [20] studied the shape and the density of ICHs and showed that large ICHs were significantly more irregular in shape, heterogeneous in density, and had greater growth. They concluded that density heterogeneity independently predicted ICH growth. Density heterogeneity in that study correspond what Zimmerman et al. [8] and we in this study call “Swirl sign”. Kim et al. [5] showed that swirl sign among other predictors was associated with increased mortality but on multivariate analysis only contrast extravasation on CT-angiography (spot sign) independently predicted mortality and hematoma growth.

To the best of our knowledge, there is no previous report on the association between the occurrence of swirl sign in ICH and the functional outcome. Our study showed that swirl sign is an ominous sign as it was associated with increased rate of death during the first month, unfavorable functional outcome at three months and was assocaited with serious radiological signs such as midline shift and intraventricular haemorrhage. Dowlatshahi et al. showed that smaller hematomas are associated with less hematoma expansion and good outcome [21]. In our study, ICHs with no swirl sign was smaller than those with swirl sign, which might indirectly indicate lower risk of hematoma expansion in the absence of swirl sign.

Patients arriving to hospital later than 24 h after onset of symptoms showed swirl sign less frequently than patients arriving earlier than 2 h indicating that those patients survived the phase of active bleeding and upon arrival after 24 h their haemorrhages were often solid and clotted. Identification of patients with ongoing haemorrhage is of utmost importance in order to provide early and appropriate care and treatment. Apart from care in acute stroke unit, surgery in selected cases is so far the only recommended treatment. However, several ongoing pharmacological trials have focus on limiting hematoma growth in the acute phase [22].

Among the shortcomings of this study were its retrospective nature and the lack of the consecutive CT follow-up that allow studying the hematoma expansion. However, our study showed that ICHs with swirl sign exhibited larger volume than those with no swirl sign. An important potentially confounding variable was the lack of data about the time between arrival to hospital and the CT scan as we in this study only reported the time between the onset of stroke and arrival to hospital. However, our hospital has well established emergency routines for patients presenting with stroke. In a recent report from our institution, the median value for the time between arrival to hospital and CT-scan was 21 min (mean 27) [23].

As midline shift, intraventricular involvement and large haemorrhage volume have in our study shown to occur more frequently among patients with swirl sign and as these signs has previously proved to be a poor prognostic signs [4,24,25], radiologists should be learned to search for and report this sign during the work-up of ICH.

## Conclusion

This study showed that swirl sign in ICHs was an independent predictor of death at one month and unfavorable functional outcome at three months. Thus, swirl sign is an ominous sign and we recommend identification of this sign in cases of ICHs.

## Competing interests

The authors declare that they have no competing interests.

## Authors’ contributions

ES has contributed to analysis and interpretation of data, drafting and revision of the manuscript critically for important intellectual content, and has given her final approval of the version to be published. EZ has contributed to conception and design of the study, acquisition of data, data analysis, drafting and revision of the manuscript critically for important intellectual content, and has given her final approval of the version to be published. MB has contributed to acquisition of data, analysis and interpretation of data, revision of the manuscript critically for important intellectual content and has given his final approval of the version to be published. KAK has contributed to conception and design of the study, acquisition of data, analysis and interpretation of data, drafting the manuscript and has given his final approval of the version to be published. All authors read and approved the final manuscript.

## Pre-publication history

The pre-publication history for this paper can be accessed here:

http://www.biomedcentral.com/1471-2377/12/109/prepub
